# To what extent are the mental health needs of children and young people (aged 11-18) in care being supported?

**DOI:** 10.23889/ijpds.v7i3.1778

**Published:** 2022-08-25

**Authors:** Carl Marincowitz

**Affiliations:** 1 Cardiff University

## Objectives

The objective of this research is to provide updated data regarding the prevalence of mental health disorders within the population of care-experienced young people (aged 11-18) in Wales. Furthermore, pathways to referrals to specialist mental health services will be investigated, as well as subsequent engagement with these services.

## Approach

Ten years of population-level routinely collected data that is available within an anonymised, information linkage databank will be used. Eight datasets will be examined; one that will provide information on the population of interest, three comparison groups, and four that contain clinical data.

Statistical analyses will be conducted to investigate the prevalence of mental health disorders among care-experienced young people. Read codes, which are used to record clinical summary information, will be used to identify poor mental health.

Free-text searches will also be conducted on all health-related datasets to improve identification of outcomes.

## Results

Expected results include revised data of the mental health needs of care-experienced young people, with prevalence in this group compared to children and young people receiving professional support in the general population. Prevalence will be looked at in relation to changes across time, as well as across demographic characteristics, including gender, age, ethnicity, local authority, placement type, and number of moves within care.

Outcomes after diagnosis of a mental health disorder will be analysed and, if an onward referral is made, the time from referral to service access will be shown, both at a national level and for local authorities. Number of referrals made at each local authority will also be shown. Lastly, young people’s engagement with mental health services will be studied.

## Conclusion

It is anticipated that findings will highlight the specific mental health needs of this group including contextual information about and in response to these needs. Findings are likely to have policy relevance by informing mental health inequality targets and strategic planning for improving outcomes for care-experienced young people.

